# Effects of Amikacin Liposome Inhalation Suspension and Amikacin Resistance Development in Patients With Refractory *Mycobacterium avium* Complex Pulmonary Disease

**DOI:** 10.1093/ofid/ofaf118

**Published:** 2025-03-01

**Authors:** Yu Kurahara, Shiomi Yoshida, Asami Osugi, Yuya Tanaka, Takehiko Kobayashi, Toshiharu Mitsuhashi, Yohei Kawasaki, Satoshi Mitarai, Kazunari Tsuyuguchi

**Affiliations:** Department of Internal Medicine, NHO Kinki Chuo Chest Medical Center, Osaka, Japan; Department of Infectious Diseases, NHO Kinki Chuo Chest Medical Center, Osaka, Japan; Clinical Research Center, NHO Kinki Chuo Chest Medical Center, Osaka, Japan; Clinical Research Center, NHO Kinki Chuo Chest Medical Center, Osaka, Japan; Department of Mycobacterium Reference and Research, Research Institute of Tuberculosis, Japan Anti-Tuberculosis Association, Tokyo, Japan; Department of Internal Medicine, NHO Kinki Chuo Chest Medical Center, Osaka, Japan; Clinical Research Center, NHO Kinki Chuo Chest Medical Center, Osaka, Japan; Center for Innovative Clinical Medicine, Okayama University Hospital, Okayama, Japan; Department of Biostatistics, Graduate School of Medicine, Saitama Medical University, Saitama, Japan; Department of Mycobacterium Reference and Research, Research Institute of Tuberculosis, Japan Anti-Tuberculosis Association, Tokyo, Japan; Department of Internal Medicine, NHO Kinki Chuo Chest Medical Center, Osaka, Japan; Department of Infectious Diseases, NHO Kinki Chuo Chest Medical Center, Osaka, Japan; Clinical Research Center, NHO Kinki Chuo Chest Medical Center, Osaka, Japan

**Keywords:** amikacin liposome inhalation suspension, *Mycobacterium avium* complex, noncavitary-nodular bronchiectatic, amikacin resistance, *rrs* mutation

## Abstract

**Background:**

Amikacin liposome inhalation suspension (ALIS) is key for treating refractory *Mycobacterium avium* complex pulmonary disease (MAC-PD). However, microbiological efficacy by subtype remains unknown. The frequency and mechanism of amikacin (AMK) resistance during ALIS administration are also unclear.

**Methods:**

We retrospectively analyzed data from refractory MAC-PD patients who received ALIS for at least 6 months as an adjunct to guideline-based therapy at the NHO Kinki Chuo Chest Medical Center. We investigated the efficacy of ALIS and analyzed gene expression and the frequency of AMK resistance.

**Results:**

We enrolled 44 patients (median age, 72.0 years): 19 (43.2%) with the noncavitary nodular bronchiectatic (NC-NB) subtype and 25 (56.8%) with the cavitary subtype. Overall, sputum culture conversion was 56.8% (25/44): 84.2% (16/19) in the NC-NB subtype and 36.0% (9/25) in the cavitary subtype (*P* = .001). During intermittent dosing, conversion occurred in 50.0% (9/18). In patients with C-reactive protein (CRP) ≥1 mg/dL, cavitary subtype, and clarithromycin (CLM) resistance, the risk ratio for persistently positive cultures was 10.81 (95% CI, 1.66–70.40) compared with those with CRP <1 mg/dL, NC-NB subtype, and CLM susceptibility. Of all participants, 15.9% (7/44) had isolates with AMK resistance (minimum inhibitory concentration ≥128 µg/mL), and of these 71.4% (5/7) had *rrs* mutations.

**Conclusions:**

Regimens that included ALIS achieved higher culture conversion in NC-NB than cavitary MAC-PD cases. High CRP levels, cavitary disease, and CLM resistance predicted persistent culture positivity. AMK resistance acquired during ALIS administration may limit treatment options for refractory MAC-PD.

## Teaser

ALIS efficacy in 44 refractory MAC-PD patients was higher in nodular bronchiectatic (84.2% culture conversion) than cavitary (36.0%) subtypes. High CRP levels, cavitary disease, and clarithromycin resistance predicted poor outcomes. 15.9% of patients developed amikacin resistance, mostly through *rrs* mutations.

## Graphical Abstract

**Graphical Abstract:**
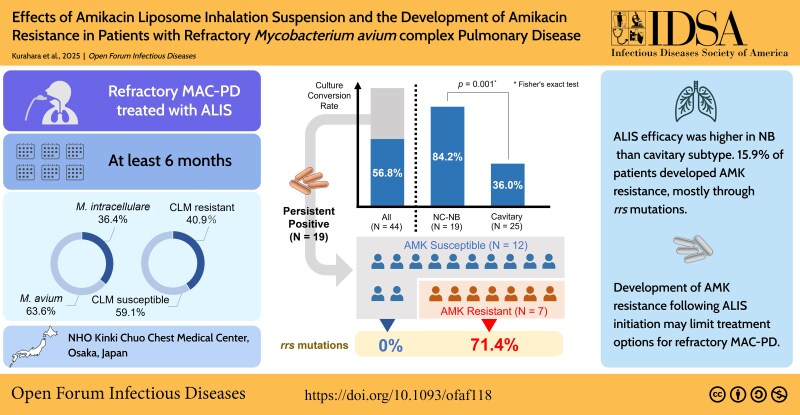
This graphical abstract is also available at Tidbit: https://tidbitapp.io/tidbits/effect-of-amikacin-liposome-inhalation-on-culture-conversion-and-development-of-amikacin-resistance-in-patients-with-cavitary-and-noncavitary-nodular-bronchiectatic-subtypes-of-refractory-mycobacterium-avium-complex-pulmonary-disease.

*Mycobacterium avium* complex (MAC) is the most isolated strain of nontuberculous mycobacteria (NTM) responsible for pulmonary infections worldwide [1, 2]. Supplementing multidrug regimens that include rifampin (RIF), ethambutol (EMB), and a macrolide with intravenous amikacin (AMK) is recommended for treating advanced or severe MAC pulmonary disease (MAC-PD) [3]. Amikacin liposome inhalation suspension (ALIS) is an inhaled antimycobacterial agent developed for refractory MAC-PD. ALIS can ensure high concentrations of AMK in the lung tissue and alveoli while minimizing systemic exposure [4]. The American Thoracic Society (ATS), European Respiratory Society (ERS), European Society of Clinical Microbiology and Infectious Diseases (ESCMID), and Infectious Diseases Society of America (IDSA) Clinical Practice Guidelines [3] recommend adding ALIS to treatment regimens for patients with MAC-PD who fail to achieve culture conversion after at least 6 months of conventional guideline-based therapy (GBT).

In the CONVERT study [5], combining ALIS with conventional GBT achieved higher culture conversion rates than GBT alone. Cavities are a risk factor for progression and higher mortality in MAC-PD [6–9]. However, there is little evidence regarding the effectiveness of ALIS against the cavitary subtype of MAC-PD, and it is unclear whether the effectiveness of ALIS–conventional GBT combination therapy is affected by the presence of a cavity. There is also concern that ALIS exposure may increase the number of AMK-resistant strains [10]. Antimicrobial resistance in MAC-PD represents a significant clinical challenge that substantially impacts patient outcomes, but real-world data on AMK minimum inhibitory concentration (MIC) remain scarce.

We aimed to fill these critical gaps by analyzing the effects of ALIS on real-world MAC-PD patients, focusing specifically on therapeutic outcomes within distinct subtypes. We also examined the MIC of AMK and resistant gene mutations in MAC-PD patients with persistently positive cultures.

## METHODS

### Study Design and Setting

We retrospectively analyzed data from refractory MAC-PD patients who initiated ALIS treatment at the NHO Kinki Chuo Chest Medical Center between August 2021 and December 2023, with all patients receiving treatment for a minimum of 6 months. Patient enrollment was completed in June 2024, with follow-up continuing through September 2024 to ensure adequate assessment of culture conversion status. For patients still in follow-up at the time of analysis, data were censored as of September 2024, which was the final data collection date. MAC-PD was diagnosed according to ATS/ERS/ESCMID/IDSA guidelines [3].

ALIS was used for patients with refractory MAC-PD, defined as those who failed to achieve negative culture conversion after at least 6 months of conventional GBT. We excluded the following cases: those with AMK MICs ≥128 µg/mL, those who could not undergo appropriate sputum testing for various reasons including early death, and those who were prescribed ALIS based solely on clinical and radiological assessment of refractory disease. Enrollment was allowed regardless of previous AMK or other aminoglycoside use. Intermittent ALIS administration was permitted at the discretion of the attending physician depending on adverse effects. Intermittent ALIS administration was defined as receiving ≤80% of the prescribed vials over a period of ≥3 months. For example, in a typical prescription cycle of 35 days, patients should receive 3 prescriptions (105 vials) over a 3-month period. If a patient received ≤84 vials (≤80% of the expected 105 vials) during this period, it was classified as intermittent administration.

We collected the following data from medical records: patient demographics, laboratory data, radiographic subtype (noncavitary-nodular bronchiectatic [NC-NB] vs cavitary subtype [fibrocavitary subtype and/or cavitary-NB subtype]), treatment regimen for MAC-PD at ALIS initiation, culture conversion, and adverse effects. A 590-mg dose of ALIS (ARIKAYCE, Insmed, Bridgewater, NJ, USA) was inhaled once daily using the LAMIRA nebulizer system (PARI, Midlothian, VA, USA) according to the manufacturer's instructions. The radiographic subtype was determined by the treating physician and subsequently reviewed by our study team. Cavity size was determined by measuring the longest diameter on axial images. The largest internal cavity diameter at confirmation was recorded. We used a C-reactive protein (CRP) cutoff value of 1.0 mg/dL [11].

### Microbiological Examinations

Sputum samples for acid-fast bacillus (AFB) testing were cultured in either a *Mycobacterium* Growth Indicator Tube (Becton, Dickinson and Company, Sparks, MD, USA) or 2% Ogawa medium (Serotec, Sapporo, Japan). Isolates were identified as distinct NTM species using GENECUBE (Toyobo, Osaka, Japan). We determined the MICs of each antimycobacterial agent via broth microdilution with Eiken dry plates (Eiken Chemical, Tokyo, Japan) or BrothMIC SGM (Kyokuto Pharmaceutical Industrial, Tokyo, Japan), according to the Clinical and Laboratory Standards Institute (CLSI) Standard M24, 3rd Edition [12]. MIC was defined as the lowest drug concentration at which no visual growth was observed. AMK resistance was defined as MIC ≥128 μg/mL, which is the CLSI-recommended breakpoint for ALIS. Isolates were considered macrolide-susceptible if the clarithromycin (CLM) MIC was ≤8 µg/mL and macrolide-resistant if the MIC was ≥32 µg/mL. Culture conversion was defined as having 3 consecutive monthly negative sputum sample results for MAC [13]. Culture conversion was assessed at least 6 months after the initiation of ALIS.

For isolates from patients whose sputum cultures remained positive after ALIS treatment, both phenotypic drug susceptibility testing (DST) and whole-genome sequencing were performed. Genotypic DST was performed by screening for mutations in known genetic resistance markers such as 16S rRNA (*rrs*) [14, 15]. Overexpression of AMK resistance–related genes was tested by analyzing aminoglycoside-modifying enzyme (*eis*) and the multidrug efflux transporter (*tap*) gene expression. These analyses were performed at the Research Institute of Tuberculosis (Supplementary Methods) [16–21]. Sequence data are available in Genbank under accession number PRJNA1189166.

### Statistical Methods

For continuous variables (age, body mass index [BMI], disease duration, CRP levels, and number of affected lobes), we used Mann-Whitney *U* tests. For categorical variables (sex, MAC species, CLM resistance, history of aminoglycoside administration, intermittent ALIS administration, and culture conversion), we used Fisher exact tests. We used the Fisher-Freeman-Halton test for categorical variables with 3 parameters (smoking history). We hypothesized that high CRP levels (≥1 mg/dL vs <1 mg/dL), radiographic subtype, and CLM resistance interact to result in a composite risk for not achieving culture conversion. Accordingly, we created an 8-level variable by combining these 3 binary variables as the exposure variable. We used a modified Poisson regression model to estimate the risk ratios (RRs) for not achieving culture conversion and their corresponding 95% CIs. In addition to crude analyses, we conducted an analysis adjusted for potential confounders (age, sex, and BMI). A *P* value < .05 was considered statistically significant. All statistical analyses were performed using Stata, version 18.5 (StataCorp, College Station, TX, USA).

This study was approved by the institutional review board of the NHO Kinki Chuo Chest Medical Center (#795; approval date: May 16, 2024; with approved protocol amendments). Patients and their families had the right to refuse participation.

## RESULTS

### Clinical Characteristics

We screened 64 patients with MAC-PD who initiated ALIS therapy at the NHO Kinki Chuo Chest Medical Center from August 2021 to December 2023. We excluded 20 patients for the following reasons: missing data (n = 9, including 1 death), treatment discontinuation <6 months (n = 9, including 1 death), and mixed infection with other mycobacteria (n = 2). The reasons for discontinuing treatment before completing 6 months were adverse effects (n = 7), poor systemic condition preventing inhalation therapy (n = 1), and financial difficulties (n = 1).

The remaining 44 patients (median age [interquartile range {IQR}], 72.0 [68.75–75.0] years; predominantly female [84.1%]) were included in our cohort (Table 1). Of these, 19 (43.2%) had the NC-NB subtype, and 25 (56.8%) had the cavitary subtype. Sixteen patients (36.4%) had a history of prior aminoglycoside administration, all occurring >6 months before ALIS treatment. The median serum CRP level at baseline (IQR) was 0.63 (0.15–2.73) mg/dL (normal value ≤ 0.3 mg/dL), with significantly higher levels in the cavitary subtype than the NC-NB subtype (2.56 vs 0.19 mg/dL; *P* = .001). The median diameter of cavities (IQR) was 2.2 (1.6–3.4) cm in the cavitary subtype. Common concomitant medications were macrolides such as CLM or azithromycin, EMB, RIF, and fluoroquinolones such as sitafloxacin or moxifloxacin.

**Table 1.** Clinical Parameters of MAC-PD Patients Treated With ALIS (n = 44)

Factors	All (n = 44)	NC-NB Subtype (n = 19)	Cavitary Subtype (n = 25)	*P* Value
Sex, male/female	7/37	1/18	6/19	.122
Age, median (IQR), y	72.0 (68.75–75.0)	73.0 (70.5–75.0)	71.0 (65.5–74.5)	.876
Body mass index, median (IQR), kg/m^2^	18.0 (16.7–19.4)	18.1 (17.0–19.5)	16.9 (16.4–19.8)	.352
Smoking history, No. (%)	…	…	…	.324
Current smoker	1 (2.3)	1 (5.3)	0 (0)	
Ever smoker	4 (9.1)	1 (5.3)	3 (12.0)	
Never smoker	39 (88.6)	17 (89.5)	22 (88.0)	
Disease duration, median (IQR), y	6.9 (5.1–11.9)	7.2 (5.1–12.3)	6.9 (5.3–11.8)	.912
CRP, median (IQR), mg/dL	0.63 (0.15–2.73)	0.19 (0.10–0.40)	2.56 (1.05–4.89)	0.001
Affected lobes, median (IQR)	3 (2–4)	2 (2–3)	4 (2.5–4)	.003
MAC species, No. (%)	…	…	…	1.000
*M. avium*	28 (63.6)	12 (63.2)	16 (64.0)	
*M. intracellulare*^a^	16 (36.4)	7 (36.8)	9 (36.0)	
CLM resistance at ALIS initiation, No. (%)	18 (40.9)	4 (21.1)	14 (56.0)	.036
History of aminoglycoside administration, No. (%)	16 (36.4)	4 (21.1)	12 (48.0)	.124
Intermittent ALIS administration	…	…	…	
At any time during treatment, No. (%)	18 (40.9)	7 (26.8)	11 (44.0)	.547
Beyond 6 mo after treatment, No. (%)	10 (22.7)	4 (21.1)	6 (24.0)	1.000
Baseline medications at the start of ALIS, No. (%)	…	…	…	–
CLM	15 (34.1)	4 (21.1)	11 (44.0)	
AZM	24 (54.5)	12 (63.2)	12 (48.0)	
EMB	32 (72.7)	13 (68.4)	19 (76.0)	
RIF	24 (54.5)	8 (42.1)	16 (64.0)	
STFX	22 (50.0)	6 (31.6)	16 (64.0)	
MFLX	1 (2.3)	0	1 (4.0)	
CFZ	1 (2.3)	0	1 (4.0)	
Culture conversion	25 (56.8)	16 (84.2)	9 (36.0)	.001
Acquired AMK resistance among those who did not achieve culture conversion	7/19	2/3	5/16	.523

Abbreviations: ALIS, amikacin liposome inhalation suspension; AMK, amikacin; AZM, azithromycin; CFZ, clofazimine; CLM, clarithromycin; CRP, C-reactive protein; EMB, ethambutol; IQR, interquartile range; MAC, *Mycobacterium avium* complex; MFLX, moxifloxacin; MIC, minimum inhibitory concentration; NC-NB, noncavitary nodular bronchiectatic; RIF, rifampicin; STFX, sitafloxacin.

^a^Genomic analysis identified 1 strain as *M. chimaera*.

The median duration (IQR) of ALIS administration (cf: 1070 days from ALIS launch to the end of the registration period) was 421.5 (258.25–578.0) days, and the median total observation period (including the 3-month post-treatment observation period) was 511.5 days. Overall, 40.9% (18/44) of the patients underwent intermittent administration at some point during treatment, mainly because of dysphonia. These 18 patients switched from daily administration to intermittent administration at a median (IQR) of 15.5 (10.75–20.75) days. Six months after initiation of ALIS, 10 patients (22.7%) were still undergoing intermittent administration.

### Adverse Events

Mild dysphonia occurred in 21 of the 44 patients (47.7%) (Table 2). Among these 21 patients, the median time to dysphonia onset (IQR) was 11.0 (7.5–15.0) days. Of these patients with dysphonia, 16 (76.2%) proceeded to intermittent ALIS administration, accounting for most of the 18 total cases of intermittent administration in our cohort. While symptoms generally improved within ∼1 month, the response was heterogeneous—some patients showed gradual improvement, while others experienced recurrence when the dose was increased. In our cohort, 8 patients (50.0%) wished to continue intermittent administration even after their dysphonia was controlled. Dysphonia was managed through a combination of dequalinium lozenges, antiseptic gargles, and adjustment of the timing of ALIS administration [22]. Cough and dyspnea were observed in 5 patients (11.4%); however, these symptoms were mild and did not interfere with ALIS continuation. Oropharyngeal pain was reported in 6 patients (13.6%) and was managed with dequalinium lozenges and antiseptic gargles. Fatigue was reported in 4 patients (9.1%). For those experiencing daytime fatigue, ALIS administration was shifted to evening hours. Mild ototoxicity was documented in 2 patients (4.5%), with no significant impact on treatment continuation. The cohort did not include patients who dropped out during treatment due to adverse effects. Among the 20 patients excluded from the analysis, 1 developed hypersensitivity pneumonitis and 5 experienced aphonia; all of these patients discontinued treatment after a brief period.

**Table 2.** Adverse Effects of ALIS (n = 44)

Adverse Effect, No. (%)	All (n = 44)
Dysphonia	21 (47.7)
Cough	5 (11.4)
Dyspnea	5 (11.4)
Oropharyngeal pain	6 (13.6)
Fatigue	4 (9.1)
Digestive symptom	1 (2.3)
Dizziness or vertigo	2 (4.5)
Tinnitus	1 (2.3)
Hearing loss^a^	1 (2.3)

Abbreviation: ALIS, amikacin liposome inhalation suspension.

^a^Hearing loss includes the preferred terms hypoacusis, deafness neurosensory, and deafness.

### Microbiological Outcome

**Figure 1.**
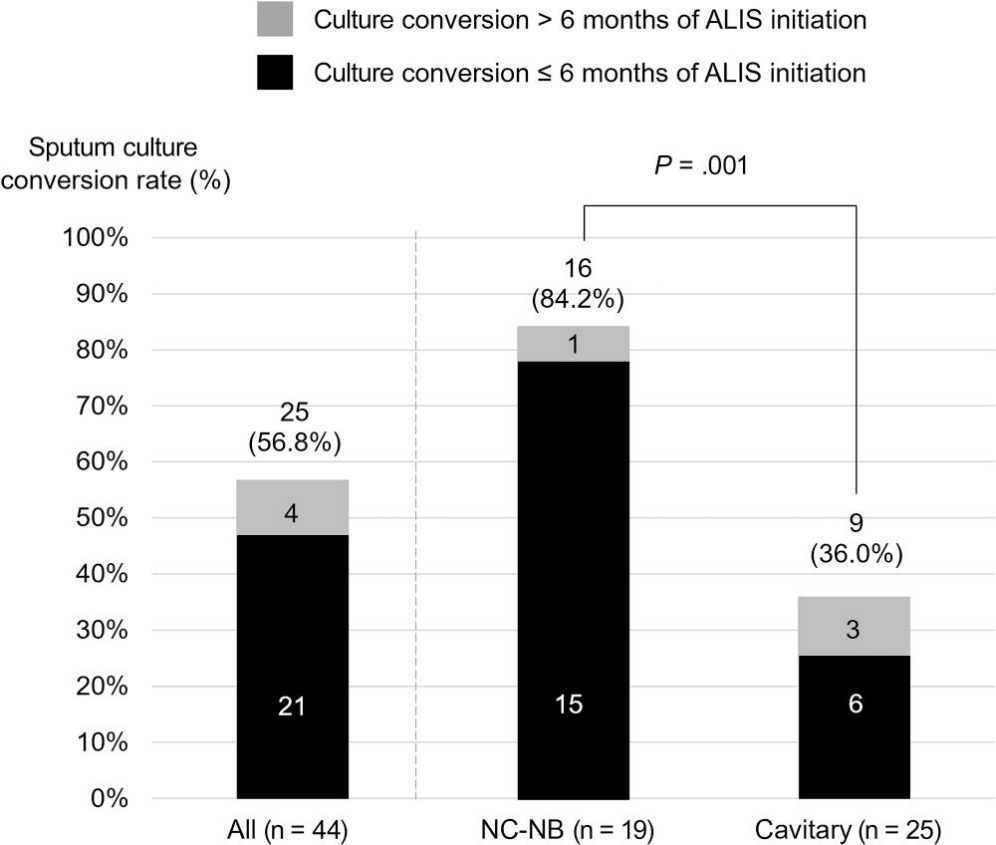
Sputum culture conversion rate (%). Gray areas indicate culture conversion occurring beyond 6 months after ALIS initiation, while black areas indicate culture conversion up to 6 months after ALIS initiation. Abbreviations: ALIS, amikacin liposome inhalation suspension; NC-NB, noncavitary nodular bronchiectatic.

The overall sputum culture conversion rate was 56.8% (25/44), with 84.2% (16/19) in the NC-NB subtype and 36.0% (9/25) in the cavitary subtype (*P* = .001) (Figure 1). Of the 25 cases with culture conversion, 18 cases (72.0%) achieved conversion within 6 months of ALIS initiation. The median time to culture conversion (IQR) was 147.0 (136.0–190.0) days. Of the 25 cases with culture conversion, 17 cases (68.0%) received ALIS treatment for >12 months. However, no patients achieved initial culture conversion beyond 12 months after treatment. Culture conversion was observed in 9 of the 18 patients (50.0%) who received intermittent dosing.

As of June 2024, 11 of the 44 patients had completed treatment. Among them, 8 patients achieved culture conversion, with their final ALIS treatment duration reaching a median (IQR) of 522.0 (435.0–615.0) days. Treatment was discontinued in 3 patients without achieving culture conversion after discussion with the patients. As of September 2024, no cases of reversion were observed among the 25 patients who achieved culture conversion.

Compared with patients who were CLM-susceptible, had the NC-NB subtype, and had CRP <1 mg/dL (reference group), patients who were CLM-susceptible, had the cavitary subtype, and had CRP ≥1 mg/dL had a risk ratio (RR) of 8.47 (95% CI, 1.22–58.87) for persistently positive cultures. Additionally, patients who were CLM-resistant, had the cavitary subtype, and had CRP ≥1 mg/dL had a risk ratio of 10.81 (95% CI, 1.66–70.40) (Table 3).

**Table 3.** Risk Ratios^a^ for Persistently Positive Cultures by CRP Level, Radiographic Subtype, and CLM Susceptibility

Factors			Culture Conversion		Nonadjusted Model		Adjusted Model^b^	
			Yes, No. (%)	No, No. (%)	RR (95% CI)	*P* Value	RR (95% CI)	*P* Value
CRP (−)	NC-NB	CLM S	12 (92.3)	1 (7.7)	1.00 (reference)	-	1.00 (reference)	-
CRP (−)	NC-NB	CLM R	3 (75.0)	1 (25.0)	3.25 (0.25–42.21)	0.368	3.24 (0.24–44.16)	.377
CRP (−)	Cavitary	CLM S	5 (71.4)	2 (28.6)	3.71 (0.39–34.98)	0.252	3.86 (0.39–37.65)	.246
CRP (−)	Cavitary	CLM R	1 (100)	0 (0)	NA	-	NA	-
CRP (+)	NC-NB	CLM S	1 (100)	0 (0)	NA	-	NA	-
CRP (+)	NC-NB	CLM R	0 (0)	1 (100)	NA	-	NA	-
CRP (+)	Cavitary	CLM S	2 (28.6)	5 (71.4)	9.28 (1.30–66,07)	0.026	8.47 (1.22–58.87)	.031
CRP (+)	Cavitary	CLM R	1 (10.0)	9 (90.0)	11.70 (1.72–79.45)	0.012	10.81 (1.66–70.40)	.013

Abbreviations: BMI, body mass index; CLM, clarithromycin: CRP, C-reactive protein; NA, not available; NC-NB, noncavitary nodular bronchiectasic subtype; R, resistant; RR, risk ratio; S, susceptible.

^a^Risk ratios were calculated using “CRP < 1.0 mg/dL, NC-NB type, and CAM-susceptible” as the reference group. They were not calculated for groups with only 1 case.

^b^The adjusted model was adjusted for age, sex, and BMI.

### AMK Resistance and *Rrs* Mutation

The MIC of AMK was measured in all 19 patients with persistently positive cultures who had received ALIS treatment for >6 months. One patient who had completed ALIS treatment switched to a distant clinic for follow-up care, making it impossible to obtain samples for analysis. Therefore, we conducted gene expression analysis on samples from the remaining 18 cases. We repeated AFB culturing at a median (IQR) of 277.0 (229.5–524.5) days after ALIS initiation in these patients.

Of the 19 patients with persistently positive cultures, we observed development of AMK resistance (MIC ≥ 128 µg/mL) in 7 (36.8%), representing 15.9% (7/44) of our total sample (Supplementary Table 1). All resistant isolates except 1 exhibited MIC ≥256 µg/mL. Of the patients with persistent positive cultures, 71.4% of AMK-resistant cases but only 8.8% of AMK-susceptible cases were cavitary (*P* = .0096) (Table 4). There was no significant difference in the proportion of intermittent ALIS administration between AMK-resistant and AMK-susceptible cases (*P* = 1.000).

**Table 4.** AMK Susceptibility of MAC-PD Patients With Persistent Positive Cultures After ALIS Treatment (n = 19)

Factors	AMK Susceptible (n = 12)	AMK Resistant (n = 7)	*P* Value
Sex, male/female	3/9	2/5	1.000
Age, median (range), y	70.5 (58.0–78.0)	71.0 (64.0–79.0)	1.000
Radiographic subtype, No. (%)	…	…	.0096
NC-NB subtype	11 (91.2)	2 (28.6)	
Cavitary subtype	1 (8.8)	5 (71.4)	
Body mass index, median (range), kg/m^2^	18.0	16.7 (15.1–18.9)	.128
Smoking history, No. (%)	…	…	1.000
Current smoker	0 (0)	0 (0)	
Ever smoker	1 (8.3)	1 (14.3)	
Never smoker	11 (91.7)	6 (85.7)	
Disease duration, median (range), y	6.8 (5.0–19.8)	7.0 (4.0–18.3)	.833
CRP, median (range), mg/dL	4.01 (0.16–9.8)	1.56 (0.12–3.07)	.120
Affected lobes, median (range)	4 (3–5)	4 (2–5)	.959
MAC species, No. (%)	…	…	1.000
*M. avium*	8 (66.7)	4 (57.1)	
*M. intracellulare*	3 (25.0)	2 (28.6)	
*M. chimaera*	1 (8.3)	1 (14.3)	
*Rrs* mutations,^a^ No. (%)	0 (0)	5 (71.4)	.0018
Position 1408	0 (0)	4 (80.0)	
Position 1482	0 (0)	1 (20.0)	
CLM resistance at ALIS initiation, No. (%)	6 (50.0)	5 (71.4)	.633
History of aminoglycoside administration, No. (%)	8 (66.7)	4 (57.1)	1.000
Intermittent ALIS administration	…	…	
At any time during treatment, No. (%)	6 (50.0)	3 (42.3)	1.000
Beyond 6 mo after treatment, No. (%)	2 (16.7)	2 (28.6)	.603

Abbreviations: ALIS, amikacin liposome inhalation suspension; AMK, amikacin; CLM, clarithromycin; CRP, C-reactive protein; MAC, *Mycobacterium avium* complex; NC-NB, noncavitary nodular bronchiectatic.

^a^ *Escherichia coli* numbering.

We identified *rrs* mutations in 5 of 7 (71.4%) AMK-resistant isolates (MIC ≥ 128 µg/mL). Four had mutations at position 1408, and 1 had a mutation at position 1491 (*Escherichia coli* numbering) (Supplementary Figure 1). All 5 cases with *rrs* mutations exhibited cross-resistance with preexisting CLM resistance. Genome analysis of the 18 cases we had samples for revealed 3 instances of reinfection, based on distance between single nucleotide polymorphisms (SNPs) (Supplementary Table 2) and mixed infections. All 3 reinfection cases showed resistance to AMK, with *rrs* mutations in 2 of them. Of the other 15 cases, only 4 (26.7%) developed AMK resistance, with *rrs* mutations in 3. The DNA sequences of the *tap* and *eis* orthologs (showing 73% and 35% amino acid sequence identity, respectively, with the *M. tuberculosis* reference strain H37Rv) remained identical among isolates from the same patients.

## DISCUSSION

We found a significant difference in culture conversion rate between the NC-NB (84.2%) and cavitary (36.0%) subtypes. This disparity suggests that ALIS may be particularly effective in treating NC-NB subtype MAC-PD, possibly due to better drug penetration in noncavitary lesions. In our cohort, the median cavity diameter in the cavitary subtype was 2.2 cm. While it is generally accepted that cavities with a diameter exceeding 2 cm are associated with a higher risk of difficult culture conversion [8], 36.0% of our patients with the cavitary subtype nevertheless achieved culture conversion. This suggests that treatment may achieve a degree of efficacy even in the presence of cavities. The efficacy of ALIS treatment for newly diagnosed MAC-PD without cavities will be assessed in phase III trials (NCT04677569 and NCT04677543). Based on the present findings, administration of ALIS for the NC-NB subtype of refractory MAC-PD is more effective than for the cavitary subtype. Patients with high CRP levels, the cavitary subtype, and CLM resistance were ∼10 times more likely to have persistent positive cultures than those with low CRP levels, the NC-NB subtype, and CLM susceptibility. Although both CRP levels and CLM resistance status were associated with treatment outcomes, the consistently higher risk ratios observed in patients with cavitary subtype across different subgroups suggest that cavitary disease may be a particularly important factor in treatment response. However, given our limited sample size, these findings should be interpreted with caution. It is therefore likely that the substantial differences in culture conversion rates are attributable to differences in the patients’ disease states.

Dysphonia was the most common adverse event, affecting 47.7% of patients and leading to treatment modifications in some cases. The high rate of intermittent dosing (40.9% of patients at any time during treatment) highlights the importance of flexible treatment approaches to manage side effects and maintain long-term adherence. Intermittent dosing is often implemented in clinical practice when necessitated by dysphonia. We observed culture conversion in 9 of the 18 patients who received intermittent dosing, and intermittent dosing did not appear to be a significant risk factor for treatment failure. However, our sample size was small, and larger studies on intermittent dosing are needed to confirm this finding.

AMK resistance (MIC ≥ 128 µg/mL) emerged in 15.9% of patients after a median of 277 days of ALIS treatment. The CONVERT study [5] showed that AMK MIC >64 µg/mL emerged in 10.3% of patients following initiation of add-on ALIS treatment and emerged in 2.7% of patients receiving conventional GBT. In a study of 33 patients with *M. abscessus* pulmonary disease (MABS-PD) who were treated with ALIS, 18% of patients showed mutational AMK resistance [23]. ALIS usage may therefore have the potential to induce AMK resistance. Managing the emergence of AMK resistance is of critical importance for MAC-PD treatment. AMK-resistant strains frequently exhibit concurrent CLM resistance. A previous small study [24] reported that patients with MAC-PD and preexisting CLM resistance failed to achieve culture conversion with ALIS and that AMK resistance emerged in 2 of 3 cases. We observed cross-resistance to CLM and AMK in all cases where *rrs* mutations were identified. This cross-resistance pattern may substantially restrict the available treatment options. Generally, the primary mechanism of acquired CLM resistance in MAC is associated with mutations in 23S rRNA (encoded by *rrl*). It has been reported that all *rrs* gene mutations in NTM are accompanied by *rrl* gene mutations [25], and this cross-resistance is thought to be a common phenomenon in refractory cases. In this study, all strains with elevated AMK MICs showed high CLM MICs, suggesting a potential association between elevated AMK MICs and poorer treatment outcomes. However, as that study examined MABS, the same phenomenon may not necessarily hold true for MAC. Further, the combined elevated MICs may predispose strains to develop higher-level resistance, risking treatment failure and worse patient outcomes.

During AMK treatment for MAC-PD, resistance develops primarily through *rrs* mutations, which encode 16S rRNA [26]. In our analysis, we found that 71.4% of AMK-resistant MAC isolates demonstrated *rrs* mutations, while observing no such mutations in AMK-susceptible isolates. Previous studies have examined the relationship between AMK MICs and *rrs* mutations in mycobacterial isolates. In *M. avium* isolates, *rrs* mutations were found in all strains with MICs >64 µg/mL, while strains with MICs of 32–64 µg/mL had wild-type sequences [27]. Similarly, among MAC-PD patients with AMK MICs ≥64 µg/mL, *rrs* mutations were present in 100% of isolates with MICs ≥128 µg/mL but only in 13.0% (3/23) of isolates with an MIC of 64 µg/mL [15]. The predominant mutation was A1408G, found in 77.4% (24/31) of isolates with MICs ≥128 μg/mL, while the remaining isolates carried C1409T, G1491C, G1491T, or C1496T mutations. In MABS-PD patients receiving ALIS treatment, 6 out of 33 patients (18.2%) developed AMK MICs >64 μg/mL, all of whom had confirmed *rrs* mutations [23]. In our study, mutations at position 1408 were found in 4 of the 5 isolates. In AMK-resistant MAC isolates without *rrs* mutations, other mechanisms may contribute to low-level AMK resistance; for example, resistance may arise via *eis* and *tap*. However, no genes with sufficient homology to be considered orthologs of these resistance genes were altered in the MAC isolates. Further, 3 of 18 cases (16.7%) with persistent positive cultures showed evidence of reinfection with AMK-resistant strains. These cases had lower single nucleotide variants compared with typical NTM recurrence cases [28], suggesting an intermediate state between strain persistence and exogenous reinfection. The emergence of *rrs* mutant subclones adapting under ALIS treatment pressure may have contributed to the development of AMK resistance.

Our study has several limitations. First, as a single-center retrospective study, our findings may not be generalizable. Our higher culture conversion rate than that recorded in the CONVERT study (29.0%) [3] may reflect our cohort's larger proportion of NC-NB subtype cases (43.2%). Our culture conversion rate was comparable to that reported by Urabe et al [29]., who recorded a 58.3% conversion rate in a similar real-world setting. Second, the inclusion of patients on intermittent ALIS administration reflects real-world practice, where dosing may be adjusted in response to side effects and to ensure adherence. Notably, 50% of these patients still achieved culture conversion. Third, although the timing of phenotypic AFB testing varied due to the study's retrospective nature, no additional initial culture conversions occurred after 12 months of treatment, suggesting that the observation period was sufficient. The timing of MIC measurements and gene expression analyses also varied for persistently culture-positive cases. However, given the scarcity of real-world data on ALIS-induced AMK resistance, these findings remain clinically valuable. Finally, our results reflect Japanese clinical practice patterns, including the continuation of macrolides for their anti-inflammatory effects despite CLM resistance and a higher rate of fluoroquinolone use than in other regions.

## CONCLUSIONS

In conclusion, our study revealed a higher culture conversion rate in NC-NB subtype than in cavitary subtype refractory MAC-PD in Japanese patients. We observed AMK resistance in 15.9% of patients who received ALIS treatment for at least 6 months, and 71.4% of AMK-resistant isolates demonstrated *rrs* mutations. The development of AMK resistance after ALIS initiation may limit treatment options for refractory MAC-PD.

## Supplementary Material

ofaf118_Supplementary_Data

## Supplementary Data

Supplementary materials are available at *Open Forum Infectious Diseases* online. Consisting of data provided by the authors to benefit the reader, the posted materials are not copyedited and are the sole responsibility of the authors, so questions or comments should be addressed to the corresponding author.
