# Supplementary material for: Effects of Amikacin Liposome Inhalation Suspension and Amikacin Resistance Development in Patients With Refractory Mycobacterium avium Complex Pulmonary Disease
Source: Open Forum Infect Dis. 2025 Mar 1;12(3):ofaf118. doi: 10.1093/ofid/ofaf118 (PMC11920506; doi:10.1093/ofid/ofaf118)
Supplement: ofaf118_Supplementary_Data [file ofaf118_supplementary_data.zip › Supplementary.docx]

**-- Supplemental Materials --**

**Clinical Outcomes of Amikacin Liposome Inhalation Suspension for *Mycobacterium avium* complex Pulmonary Disease and Verification of Amikacin Resistance**

Author: Kurahara Y, Yoshida S, Osugi A, et al.

Corresponding author: Tsuyuguchi K, [tsuyuguchi.kazunari.ky@mail.hosp.go.jp](mailto:tsuyuguchi.kazunari.ky@mail.hosp.go.jp)

**Appendix A. Supplemental Methods**

-- Genome analysis

**Appendix B. Supplemental Tables**

-- Supplemental Table 1: Drug susceptibility testing of MAC before and after ALIS initiation

-- Supplemental Table 2: Distance measures of SNPs in isolates before and after ALIS treatment in MAC

**Appendix C. Supplemental Figure**

-- Supplemental Figure 1. Phenotypic and genotypic DST results in pre- and post-ALIS-exposed MAC isolates from refractory MAC-PD patients with whole genome sequencing-verified relapse (n = 15) and reinfection (n = 3)

**Appendix A. Supplemental Methods**

**Genome analysis*:**

Total genomic DNA of 40 MAC isolates were subjected to QIAseq FX DNA library kit (Qiagen, Hilden, Germany) before paired-end sequencing (2 × 150 bp) on NextSeq 550 instrument (Illumina, San Diego, CA, USA), according to the manufacturer's instructions. Samples were considered MAC isolates if >90% of sequence reads were assigned into “*Mycobacterium avium* complex (MAC)” using Kraken2 v2.1.2 [16]. Samples identified as MAC isolates were subjected to variant calling between isolates from the individual patients. Sequence reads were processed using Trimmomatic v0.39 [17] to remove low-quality reads and then mapped to a reference genome using BWA-mem v0.7.17 [18]. OCU901 was used as the reference genome since most isolates (19/40) were closest to it within the RefSeq bacteria directory v.221 using ReferenceSeeker v1.8.0 [19]. Variants were called using bcftools v1.11 “mpile up” command [20], and filtered by allele frequency >75%, mapping quality >30 and depths of high-quality bases >20 in the FORMAT column, and > 25 in the QUAL column. Isolates were considered a mixed infection of *M. avium* and *M. intracullulare* if the percentage of alignments to *M. avium* and *M. intracullulare* genomes was not high (> 90%) but intermediate (> 80%) and if any genomes in RefSeq bacteria directory v.221 have ANI < 90%. Patients were considered reinfection if SNP numbers between isolates from the same patients were > 1,000. Isolates from the same patients were considered genetically related if SNP number between isolates were < 25 [21].

*Note: References [16] to [21] can be found in the main manuscript.

ANI, average nucleotide identity; DNA, deoxyribonucleic acid; MAC, *Mycobacterium avium* complex; SNP, single nucleotide polymorphism

**Appendix B. Supplemental Tables**

**Supplemental Table 1.** DST of MAC before and after ALIS initiation

|  | Pre-ALIS MIC (before ALIS initiation) | | | | | Post-ALIS MIC (median 277.0 days after ALIS initiation) | | | | |
| --- | --- | --- | --- | --- | --- | --- | --- | --- | --- | --- |
|  | No. | S | I | R | %R | No. | S | I | R | %R |
| CLM | 44 | 18 | 0 | 16 | 36.4 | 19 | 8 | 0 | 11^a^ | 57.9 |
| AMK | 44 | 44 | 0 | 0 | 0 | 19 | 12 | 0 | 7 | 36.8 |

^a^ One patient developed resistance after ALIS treatment despite initial CLM susceptibility.

ALIS, amikacin liposome inhalation suspension; AMK, amikacin; CLM, clarithromycin; DST, drug susceptibility testing; MIC, minimum inhibitory concentration; S, susceptible; I, intermediate; R, resistant; %R, percentage of resistant isolates.

**Supplemental Table 2**. Distance measures of SNPs in isolates before and after ALIS treatment in MAC^1^

| Allele Frequency | Case A | B | C | D | E | F | G | H | I | J | K | L | M | N | O | P | Q | R |
| --- | --- | --- | --- | --- | --- | --- | --- | --- | --- | --- | --- | --- | --- | --- | --- | --- | --- | --- |
| >0.90 | 0 | 10 | 2 | 2 | 0 | 2650 | 0 | 0 | 23 | 5 | 10 | 0 | 0 | 1 | 8447 | 0 | 0 | 0 |
| >0.75 | 0 | 15 | 2 | 5 | 1 | 3107 | 0 | 0 | 24 | 6 | 22 | 0 | 0 | 1 | 8462 | 0 | 1 | 0 |

^1^Variants were called using bcftools v1.11 “mpile up” command, and filtered by allele frequency > 75% or > 90%, mapping quality > 30 and depths of high-quality bases > 20 in the FORMAT column, and > 25 in the QUAL column.

**Appendix C. Supplemental Figure 1**

**Supplemental Figure 1. Phenotypic and genotypic DST results in pre- and post-ALIS-exposed MAC isolates from refractory MAC-PD patients with whole genome sequencing-verified relapse (n = 15) and reinfection (n = 3).** Letters represent individual cases, and those with primes indicate strains that remained culture-positive after ALIS initiation. Each circle represents a patient with MAC. White and black circles denote AMK-S and AMK-R isolates from MAC-PD, respectively. Core genome SNP distances between isolates from the same patients are indicated by numbers and lines. Isolates from the same patients were considered genetically related if SNP distances between isolates were < 25. ALIS, amikacin liposome inhalation suspension; AMK, amikacin; DST, drug susceptibility testing; MAC-PD, *Mycobacterium avium* complex pulmonary disease; R, resistant; S, susceptible; SNP, single nucleotide polymorphism

ALT text: A figure showing phenotypic and genotypic DST results of MAC isolates from patients with refractory MAC-PD before and after exposure to ALIS. Each circle represents an individual patient. White circles indicate AMK-susceptible isolates, while black circles denote AMK-resistant (AMK-R) isolates. SNP distances between isolates from the same patient are represented by numbers.
